# Radiological assessment of patellar instability: comparative analysis of patients with and without instability and the impact of patellar stabilisation surgery

**DOI:** 10.1007/s00256-025-05068-0

**Published:** 2025-11-10

**Authors:** Mustafa Al-Zubaidy, Kira Faircloth, Oday Al-Dadah

**Affiliations:** 1 Translational and Clinical Research Institute, Faculty of Medical Sciences, Newcastle University, Framlington Place, Newcastle upon Tyne, NE2 4HH UK; 2 Department of Trauma and Orthopaedic Surgery, South Tyneside District Hospital, Harton Lane, South Shields, NE34 0PL UK

**Keywords:** Patellar instability, X-ray, Magnetic resonance imaging, Knee, Patellar stabilisation surgery

## Abstract

**Background:**

Patellar instability (PI) is a clinical diagnosis often complicated by symptom overlap with other knee pathologies and spontaneous relocation of the patella. Anatomical variations are key risk factors, highlighting the role of radiological investigations in assessing underlying pathology. Inconsistent measurement parameters and limited comparative data reduce diagnostic reliability. This study aimed to compare radiological parameters between patients with and without PI, and to assess how these parameters change following patellar stabilisation surgery.

**Methods:**

This case-control study compared magnetic resonance imaging (MRI) and X-ray radiological parameters between patients with recurrent PI and controls. Pre- and postoperative imaging was analysed in the PI group. Eleven validated radiological measurements, including patellofemoral joint angles and patellar height indices, were evaluated.

**Results:**

Fifty-five knees in the PI group and 50 knees in the Control group were analysed. Preoperatively, significant differences were found for all patellar height indices (*p* < 0.001) and patellofemoral measurements (*p* < 0.05), except for the congruence angle. Postoperatively, all patellar height measurements improved in the PI group (*p* < 0.05), with only the sulcus angle improving (*p* = 0.005) for patellofemoral measurements. Significant differences were observed between MRI and X-ray measurements (*p* < 0.05).

**Conclusion:**

Radiological parameters differ significantly between unstable and stable knees, with improvement following surgery. Patellar height was the most consistent marker of instability. Discrepancies between MRI and X-ray findings suggest a need for modality-specific normative values. These results reinforce the value of radiological assessment in PI and support standardisation to improve diagnostic accuracy.

## Introduction

Patellar instability (PI) is a clinical diagnosis characterised by the pathological disarticulation of the patellofemoral joint 3 or more times within a short timeframe [1]. With an estimated incidence of 5.8 cases per 100,000, increasing to 29 cases per 100,000 during adolescence, it is a common presentation in knee surgery [2]. Initial dislocations are typically managed conservatively with bracing and physiotherapy. However, persistent pain in 70% of patients and recurrence in 30–50% have prompted considerations for early surgical intervention, with the aim to prevent recurrent instability, joint damage, and potential progression to osteoarthritis [3–6]. The most prominent issue for PI patients remains a successful early diagnosis.

Difficulties remain in the diagnosis of PI due to most dislocated patellae relocating spontaneously prior to presentation [2]. Presentations can be similar in PI, patellofemoral pain, ligamentous insufficiencies, and degenerative knee conditions, often including nonspecific anterior knee pain and apprehension towards the knee’s stability. This overlap creates a complex clinical conundrum that requires further investigation to differentiate [5]. In clinical practice, an apprehension test can be performed as part of the physical examination of the knee in the outpatient clinic. In a positive result, the patient contracts the quadriceps to protect against dislocation, which strongly indicates instability. However, a negative test does not exclude malalignment [6]. Without a test offering certainty, further investigations, such as radiology, are left as the most definitive diagnostic test.

The patellofemoral joint is stabilised through the relationship between the osseous structures and the stabilising ligamentous vectors, meaning even minor anatomical variations are major risk factors in the development of instability [1, 4, 5]. These morphological differences influence the surgeries commonly used: medial patellofemoral ligament (MPFL) reconstruction, tibial tuberosity transfer (TTT), and trochleoplasty. Consequently, radiology plays a central role in clinical practice and research. In 1974, Merchant et al. [5] developed the skyline view to further visualise the joint by angling the X-ray beam at 30 degrees towards a 45-degree flexed knee. Dejour et al. [7] undertook extensive research into identifying pre-disposing anatomical factors radiographically, identifying four significant factors in PI: trochlear dysplasia, patella alta, an increased tibial tuberosity-trochlear groove (TT-TG) distance, and patella tilt. Trochlear dysplasia, characterised by a shallow or malformed trochlear groove, disrupts patellar tracking, predisposing it to lateral displacement [8]. Patella alta, defined as an abnormally high-riding patella due to an elongated patellar tendon, delays trochlear groove engagement during flexion, increasing dislocation risk, particularly in early ranges of motion [1, 9]. An elevated TT-TG distance reflects lateral malalignment of the extensor mechanism, which redirects the patellar tendon obliquely and increases the risk of subluxation [4, 10]. Patella tilt, often laterally, reduces the articulating surface area and is commonly seen alongside other structural abnormalities such as trochlear dysplasia or MPFL insufficiency [8, 10]. Together, these anatomical risk factors are important in the diagnosis of PI.

Despite this, significant variation exists between the agreed radiological modality and parameters, with the abundance of inconsistently used measurements highlighting their discrepancies [4]. This uncertainty is echoed in a meta-analysis by Smith et al. [11] recommending further study of the reliability and validity of the Insall–Salvati ratio, Caton–Deschamps, Blackburne-Peel, and TT-TG. Existing literature around PI lacks comprehensive comparisons between patients with and without PI using multiple radiographic parameters, limiting the ability to discern significant deviations from normal anatomy [12]. A systematic review by White et al. [13] highlighted the need for further validation of radiographic parameters and their roles in the assessment of patellar instability. Similarly, few studies compare radiographic changes in PI patients before and after patellar stabilisation surgery using multiple parameters, with most research focusing on clinical outcomes, recurrence rates, and patient-reported measures rather than detailed radiological evaluations [14, 15].

The primary aim of this study was to compare radiological parameters between patients with and without patellar instability. The secondary aim was to evaluate how these parameters change following patellar stabilisation surgery. The hypotheses are that radiological measurements differ significantly between stable and unstable knees and that these measurements improve postoperatively in the patellar instability group.

## Methods

### Patient cohort

**Fig. 1:**
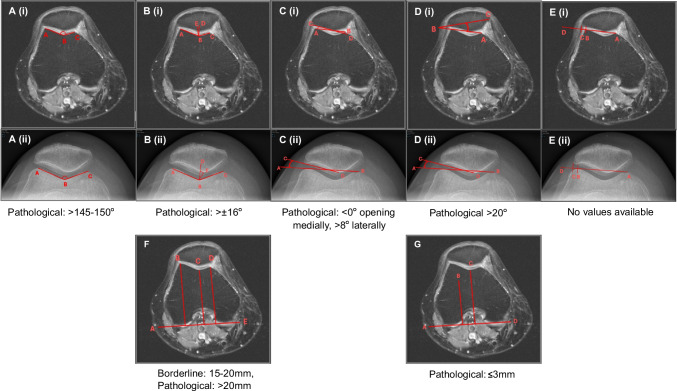
Radiological measurement methodology for patellofemoral joint measurements using axial MRI and skyline X-ray images. **A** Sulcus angle (SA): (i) MRI and (ii) X-ray: SA is angle < ABC, formed by Lines AB (lateral femoral condyle summit to the deepest trochlear groove) and BC (medial femoral condyle summit to the same point) (Brattstrom et al. [17]). **B** Congruence angle (CA): (i) MRI and (ii) X-ray: CA is angle < DBE, where Line BD bisects SA (< ABC), and Line BE extends to the lowest point of the patellar articular ridge. Medial orientation is negative, lateral is positive (Merchant et al. [18]). **C** Lateral patellofemoral angle (LPFA): (i) MRI and (ii) X-ray: LPFA is angle between Tangents AB (medial-lateral femoral condyle summits) and CD (lateral patellar facet) (Laurin et al. [16]). **D** Patella tilt angle (PTA): (i) MRI and (ii) X-ray: PTA is angle < ABC, formed by Tangents AB (femoral condyle summits) and BC (patella’s transverse axis). Lateral tilt is positive, medial tilt is negative (Grelsamer et al. [19]). **E** Lateral patella shift (LPS): (i) MRI and (ii) X-ray: LPS is calculated as (BC/AB) × 100%, where AB is femoral condylar width and BC is patellar displacement (Bito et al. [20]). **F** Trochlear depth (TD) (MRI only): TD is perpendicular distance from Line AE (posterior femoral condyles) to the lowest point of the trochlear groove, subtracted from the mean height of the medial and lateral trochlear facets (Pfirrmann et al. [21]). **G** Tibial tuberosity-trochlear groove (TT–TG) (MRI only): TT–TG is horizontal distance between the tibial tuberosity and trochlear groove, measured from perpendicular lines extending from the posterior femoral condyles (Line AD) to the tibial tuberosity (Line B) and the trochlear groove (Line C) (Dejour et al. [7])

**Fig. 2:**
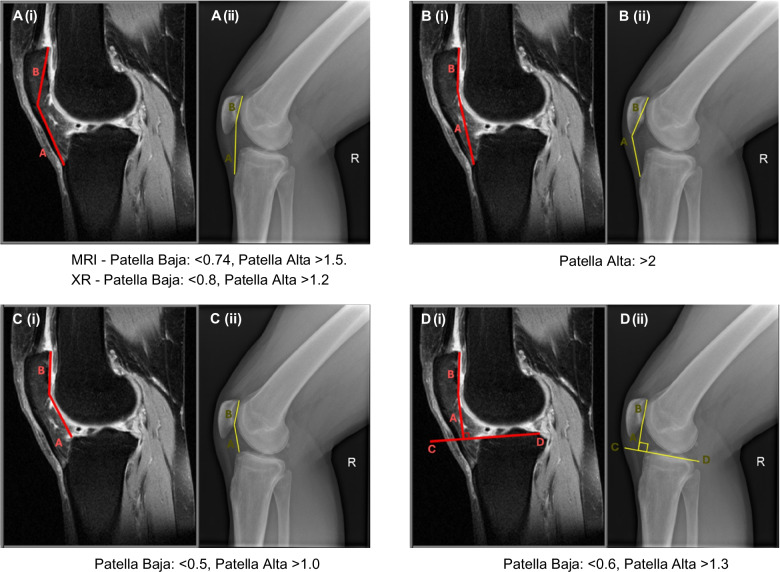
Radiological measurement methodology for patellar height measurements using sagittal MRI and lateral X-ray images. **A** Insall–Salvati ratio (IS): (i) MRI and (ii) X-ray: IS ratio is patellar tendon length (Line A, from the inferior pole of the patella to the tibial tuberosity) divided by patellar height (Line B, from the inferior to the superior pole of the patella) (Insall and Salvati [22]). **B** Modified Insall–Salvati ratio (MIS): (i) MRI and (ii) X-ray: MIS ratio is length from the inferior patellar surface to the tibial tuberosity **A** divided by patellar articular surface height **B** (Grelsamer and Meadows [23]). **C** Caton–Deschamps ratio (CD): (i) MRI and (ii) X-ray: CD ratio is distance from the inferior patellar articular surface to the tibial plateau **A** divided by patellar articular surface height **B** (Caton and Deschamps [25]). **D** Blackburne–Peel index (BP): (i) MRI and (ii) X-ray: BP index is distance from the tibial plateau to the inferior patellar articular surface **A** divided by patellar articular surface height **B** (Blackburne and Peel [24])

This was a retrospective case-control study. This study was registered with the Clinical Effectiveness Department (registration number CA10094) at the host institution. All procedures performed in studies involving human participants were in accordance with the ethical standards of the institutional research committee and with the 1964 Declaration of Helsinki and its later amendments. Patients included in the study were identified under the care of a Consultant Orthopaedic Surgeon who specialises in knee surgery. The choice of which specific surgical intervention was determined based on clinical examination and the identified underlying pathological anatomy as yielded from the radiological investigations. Patients with significant trochlea dysplasia underwent trochleoplasty, patients with marked patella alta underwent TTT (antero-medial distalisation), and patients with relatively normal patellar height and trochlea groove underwent MPFL reconstruction. This study constituted part of the second author’s Masters dissertation, from which some data points from the same cohort of patients have been used in another study that has also been submitted for publication; however, the emphasis of the data analysed herewith and the specific study aims and hypothesis are entirely different and original. Figures 1 and 2 have also been replicated in both papers to illustrate the radiological measurement techniques as they share this commonality.

The inclusion criteria for the PI group were as follows: recurrent patellar instability resulting in full dislocations, symptoms unresponsive to initial conservative treatment (i.e. activity modification, physiotherapy), completion of patellar stabilisation surgery, and availability of pre- and post-surgical radiological investigations. Inclusion criteria for the Control group consisted of patients without a history or clinical evidence of patellar instability, but with other knee pathologies (e.g. meniscus tears and loose bodies), who had undergone radiological investigations. Exclusion criteria included prior patella surgery to the index knee and the absence of knee X-rays and MRI scans of the same knee. Baseline demographic data and smoking status were collected for both groups.

### Radiological parameters

Skyline and lateral view X-rays and axial and sagittal view magnetic resonance imaging (MRI) scans were viewed on the Picture Archiving Communication System (PACS) (Centricity version 6, GE Healthcare, Chicago) by a single observer, trained by a consultant orthopaedic knee specialist. MRIs were completed preoperatively only for both the PI group and Control group. However, X-rays were completed both preoperatively and postoperatively for the PI group but only preoperatively for the Control group. Skyline view X-ray and lateral view X-ray, both with the knee in 30-degree flexion, were primarily used in the analysis, as this view is routinely used in clinical practice for patients with and without PI symptoms. MRI images (with the knee in full extension) were obtained using a 1.5-T GE Healthcare SIGNA Artist MRI scanner. The MRI sequences were obtained as per standard knee protocols used by the radiology department, which included sagittal, coronal, and axial proton density (PD) fat-saturated sequences alongside a sagittal T1-weighted sequence. The PD fat-saturated images were used for all MRI data collection. Utilising the PACS measuring tools, anatomical measurements relating to morphological risk factors of PI were collated, including 7 patellofemoral measurements and 4 patellar height measurements.

On axial MRI and skyline X-rays, radiological parameters were measured using established measurements. These include the lateral patellofemoral angle (LPFA) as described by Laurin et al. [16], sulcus angle (SA) by Brattstrom et al. [17], congruence angle (CA) by Merchant et al. [18], patella tilt angle (PTA) by Grelsamer et al. [19], and lateral patella shift (LPS) by Bito et al. [20]. Additionally, on axial MRI, trochlear depth (TD) was measured as outlined by Pfirrmann et al. [21], and the tibial tuberosity-trochlear groove (TT-TG) distance by Dejour et al. [7]. On sagittal MRI and lateral X-ray, patella height was measured using the Insall–Salvati ratio (IS) [22], the modified IS ratio (MIS) as described by Grelsamer and Meadows [23], the Blackburne–Peel index (BP) [24], and the Caton–Deschamps ratio (CD) [25]. Details and illustrations of these measurements, along with reference ranges for pathological values (including patella baja and alta), are shown in Fig. 1 (patellofemoral joint) and Fig. 2 (patellar height).

### MRI level selection

**Fig. 3:**
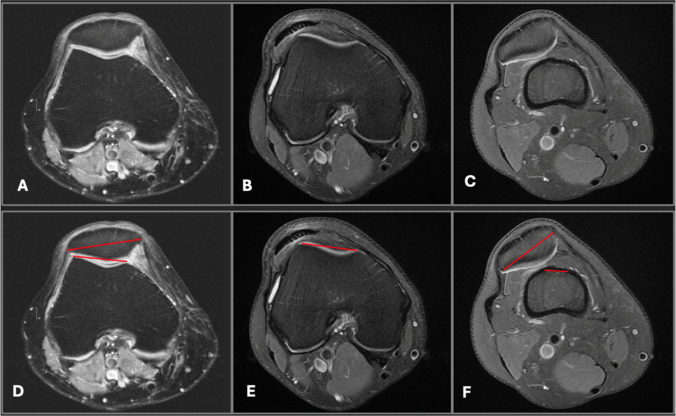
Illustration of new method for MRI image selection in patella alta. **A** Axial image of a knee with normal patellar height at the point where the posterior femoral condyles are largest. **B** Axial image of a knee with patella alta at the point where the posterior femoral condyles are largest. **C** Axial image of a knee with patella alta at the point where the patella is viewed at its largest diameter. **D **Axial image of a knee with normal patellar height at the point where the posterior femoral condyles are largest with Stefanik et al. [28 method of image selection and patella tilt angle illustrated. **E **Axial image of a knee with patella alta at the point where the posterior femoral condyles are largest illustrating the failure of Stefanik et al. [28 method to measure patella tilt angle in patella alta. **F **Axial image of a knee with patella alta at the point where the patella is viewed at its largest diameter illustrating the new method to measure the patella tilt angle

For MRI-based measurements, PD fat-saturated sequences were selected due to superior visualisation of fluid, hyaline cartilage, ligaments, and tendons compared to T1-weighted images [26]. Patellar height was assessed on sagittal images where the patella appeared at maximal length between its poles, as described by Biedert and Albrecht [27]. Initially, axial slice selection for angle measurements followed the method used in the MOST study by Stefanik et al. [28], identifying the slice with the largest posterior femoral condyles. However, in severe patella alta cases, this approach was limited as the patella and trochlear sulcus were no longer visualised in the same slice. Due to the lack of an established method to address this, a new method, outlined in Fig. 3, was developed. Angles directly involving the trochlear sulcus (TT–TG, TD, SA, and CA) continued to use Stefanik et al.’s method [28], whilst other parameters were measured on the axial slice where the patella was visualised at its maximal diameter, in line with the sagittal technique by Biedert and Albrecht [27].

### Statistical analysis

Plotted histograms with fitted curve lines, box-plots, normal Q-Q plots, and the Kolmogorov–Smirnov statistic were used to test the normality of data distribution. All X-ray (skyline view) and MRI (axial images) patellofemoral measurements displayed a normal distribution, and the relevant parametric statistical tests were used. However, almost all patellar height measurements on both X-rays (lateral view) and MRI scans (sagittal view) displayed a skewed distribution, and therefore the relevant non-parametric statistical tests were used for their data analysis. The level of statistical significance was set at *p* < 0.05. Statistical analysis was performed using SPSS for Windows version 26.0 (IBM Corp., Armonk, New York).

## Results

There were 55 knees (44 patients) included in the PI group and 50 knees (48 patients) included in the Control group. Table 1 summarises demographic data for both groups. The PI group was younger (mean age 30 vs 53 years) and had a higher proportion of females compared to the Control group. BMI, laterality, and smoking status were comparable between groups.

**Table 1:** Patient demographics for the Control group and Patellofemoral Instability group, including frequency of surgeries performed for Patellofemoral Instability group

	PI group (*n* = 55)	Control group (*n* = 50)
Mean age (years) (SD)	30 (12)	53 (15)
Gender (male to female)	16:39	29:21
Laterality (right to left)	28:27	26:24
Mean height (m) (SD)	1.63 (28.2)	1.71 (9.3)
Mean weight (kg) (SD)	88.1 (28.4)	79.9 (20.2)
Mean BMI (kg/m^2^) (SD)	28.8 (6.1)	27.3(5.7)
Smoking status (smoker to non-smoker to missing)	7:43:5	8:32:10

*PI* patellofemoral instability; *SD* standard deviation; *m* metres; *kg* kilograms; *BMI* body mass index

Table 2 shows the comparison of patellofemoral measurements on 30-degree skyline X-rays and axial MRI, and patellar height measurements on lateral X-rays and sagittal MRI between the PI group and the Control group preoperatively. For patellofemoral measurements on X-ray, the PTA (*p* < 0.001) and the SA (*p* < 0.001) were significantly greater in the PI group compared to the Control group. On MRI, all patellofemoral measurements except the CA differed significantly between groups (*p* < 0.05), with marked differences observed for LPFA, PTA, TD, and TT-TG (*p* < 0.001). For patellar height measurements on X-ray, all measurements were significantly greater (*p* < 0.001) in the PI group compared to the Control group. On MRI, all patellar height measurements were significantly greater in the PI group compared to the Control group.

**Table 2:** Comparison of patellofemoral measurements on 30-degree Skyline X-Rays and axial MRI, and also patellar height measurements on lateral X-rays and sagittal MRI between the Patellofemoral Instability group pre-surgery and the Control group

Patellofemoral measurement	PI group mean (SD)	Control group mean (SD)	*p*-value^a^	95% CI
Pre-surgery (X-ray)				
Sulcus angle (°)	143.4 (8.0)	138.3 (4.9)	<0.001*	2.4–7.8
Congruence angle (°)	−1.3 (26.1)	−2.0 (19.4)	0.892	−10.1–8.8
Lateral patellofemoral angle (°)	6.1 (6.3)	9.0 (8.1)	0.070	−0.2–6.0
Patella tilt angle (°)	17.0 (6.3)	12.8 (3.2)	<0.001*	2.2–6.2
Lateral patella shift (%)	15.9 (9.9)	13.8 (6.8)	0.236	−5.6–1.4
Pre-surgery (MRI)				
Sulcus angle (°)	138.3 (9.4)	133.8 (7.0)	0.008*	1.2–7.8
Congruence angle (°)	8.5 (33.7)	−2.2 (16.7)	0.067	−22.0–0.8
Lateral patellofemoral angle (°)	−3.9 (11.5)	3.9 (6.7)	<0.001*	4.0–11.6
Patella Tilt Angle (°)	27.0 (11.0)	20.1 (5.7)	<0.001*	−3.4–10.4
Lateral Patella Shift (%)	46.2 (43.2)	21.7 (16.7)	0.001*	10.9–37.9
Trochlear Depth (mm)	5.7 (1.9)	7.9 (1.5)	<0.001*	1.5–2.9
TT-TG (mm)	14.6 (4.9)	10.8 (3.7)	<0.001*	2.0–5.6
Patellar height measurement	PI group median (IQR)	Control group median (IQR)	*p*-value^b^	*Z*, *U*
Pre-surgery (X-ray)				
Insall–Salvati	1.4 (1.1–1.5)	1 (1–1.2)	<0.001*	−5.24, 453
Modified Insall–Salvati	1.9 (1.7–2.2)	1.6 (1.5–1.7)	<0.001*	−4.50, 417
Caton–Deschamps	1.1 (1.0–1.3)	0.9 (0.8–0.9)	<0.001*	−6.52, 279
Blackburne–Peel	1.0 (1.0–1.2)	0.8 (0.7–0.9)	<0.001*	−6.92, 217
Pre-Surgery (MRI)				
Insall–Salvati	1.4 (1.4–1.7)	1.2 (1.1–1.4)	<0.001*	−4.74, 539
Modified Insall–Salvati	2.0 (1.8–2.2)	1.8 (1.7–2.0)	0.001*	−3.47, 717
Caton–Deschamps	1.3 (1.2–1.5)	1.1 (1.0–1.2)	<0.001*	−4.78, 539
Blackburne–Peel	1.1 (1.0–1.3)	1.0 (0.9–1.1)	<0.001*	−3.90, 658

*PI* patellofemoral instability, *SD* standard deviation, *CI* confidence interval, *IQR *inter-quartile range

*Statistically significant at <0.05 level

^a^Independent sample Student’s *t*-test

^b^Mann–Whitney *U* test

Table 3 shows the comparison of patellofemoral measurements between 30-, 60-, and 90-degree skyline X-rays and MRI and patellar height measurements between lateral X-rays and sagittal MRIs preoperatively in the PI group. Between MRI and 30-degree X-rays, all measurements were significantly different. Between MRI and 60-degree X-rays, the LPS, PTA, and SA significantly differed (*p* < 0.05). Between MRI and 90-degree X-rays, the LPS and the PTA remained significantly different (*p* < 0.05). For patellar height, three measurements were shown to be significantly different between X-ray and MRI: BP (*p* = 0.023), CD (*p* < 0.001), and IS (*p* < 0.001).

**Table 3:** Comparison of patellofemoral measurements between 30-, 60-, and 90-degree skyline X-rays and axial MRI, and patella height measurements between lateral X-rays and sagittal MRIs pre-operatively in the Patellofemoral Instability group

	PI group						
Patellofemoral measurement	Pre-surgery MRI mean (SD)	Pre-surgery X-ray 30-degree mean (SD)	Pre-surgery X-ray 60-degree mean (SD)	Pre-surgery X-ray 90-degree mean (SD)	MRI vs 30-degree X-ray *p*-value^a^ (95% CI)	MRI vs 60-degree X-ray *p*-value^a^ (95% CI)	MRI vs 90-degree X-ray *p*-value^a^ (95% CI)
Sulcus angle (°)	138.3 (9.4)	143.4 (8.0)	144.0 (9.6)	145.9 (7.2)	<0.001* (3.2–6.5)	0.017* (1.3–9.9)	0.070 (−13.6–0.7)
Congruence angle (°)	8.5 (33.7)	−1.3 (26.1)	4.6 (28.0)	−0.8 (32.2)	0.041* (0.4–18.9)	0.472 (−18.7–36.9)	0.310 (−19.7–54.7)
Lateral patellofemoral angle (°)	−3.9 (11.5)	6.1 (6.3)	6.0 (4.8)	5.3 (8.8)	<0.001* (6.9–13.3)	0.075 (−23.5–1.4)	0.192 (−25.7–6.1)
Patella tilt angle (°)	27.0 (11.0)	17.0 (6.3)	15.1 (2.9)	11.5 (3.8)	<0.001* (7.0–13.3)	0.008* (6.1–29.1)	0.007* (7.8–34.9)
Lateral patella shift (%)	46.2 (43.2)	15.9 (9.9)	14.5 (7.9)	12.6 (6.6)	<0.001* (19.7–7.3)	0.036* (3.5–81.0)	0.029* (5.8–81.8)
Patellar height measurement	Pre-surgery sagittal MRI median (IQR)		Pre-surgery X-ray median (IQR)		MRI vs X-ray *p*-value^b^	*Z*	
Insall–Salvati	1.4 (1.4–1.7)		1.4 (1.1–1.5)		<0.001*	−3.49	
Modified Insall–Salvati	2.0 (1.8–2.2)		1.9 (1.7–2.2)		0.464	−0.73	
Caton–Deschamps	1.3 (1.2–1.5)		1.1 (1.0–1.3)		<0.001*	−4.00	
Blackburne–Peel	1.1 (1.0–1.3)		1.0 (1.0–1.2)		0.023*	−2.28	

*PI* patellofemoral instability, *SD* standard deviation, *CI* confidence interval, *IQR* interquartile range

*Statistically significant at <0.05 level

^a^Paired Student’s *t*-test

^b^Wilcoxon signed ranks test

Table 4 shows the comparison of patellofemoral measurements on 30-degree skyline X-rays and patellar height measurements on lateral X-rays, between pre- and postoperative for the PI group (longitudinal within-group analysis), and also the comparison of the postoperative PI group to the Control group (between-group analysis). On 30-degree skyline X-rays, there were no significant differences pre- and postoperatively for the PI group except for SA (*p* = 0.007). On 30-degree skyline X-rays, only the PTA (*p* = 0.048) was statistically different between the postoperative PI group and the Control group. On lateral X-rays, all patellar height measurements were found to be significantly improved following surgery (*p* < 0.05) in the PI group, with marked differences for BP and CD (*p* < 0.001). On lateral X-rays, all patellar height measurements were statistically different (*p* < 0.05) between the postoperative PI group and the Control group, with marked differences in IS and MIS (*p* < 0.001).

**Table 4:** Comparison of patellofemoral measurements on 30-degree skyline X-ray and patellar height measurements on lateral X-rays, between pre- and postoperative PI group (longitudinal within group analysis), and also comparison of postoperative PI group to the Control group (between group analysis)

Patellofemoral measurement	PI group pre-surgery mean (SD)	PI group post-surgery mean (SD)	Control group mean (SD)	PI pre- vs post-surgery *p*-value^a^ (95% CI)	PI post-surgery vs control *p*-value^a^ (95% CI)
Sulcus angle (°)	143.4 (8.0)	139.5 (7.9)	138.3 (4.9)	0.005* (1.0–5.4)	0.457 (−4.2–1.9)
Congruence angle (°)	−1.3 (26.1)	3.4 (24.4)	−2.0 (19.4)	0.245 (−10.1–2.7)	0.288 (−15.5–4.7)
Lateral patellofemoral angle (°)	6.1 (6.3)	9.3 (5.7)	9.0 (8.1)	0.068 (−3.9–0.2)	0.831 (−3.5–2.8)
Patella tilt angle (°)	17.0 (6.3)	14.5 (4.0)	12.8 (3.2)	0.417 (−0.8–1.9)	0.048* (0.02–3.32)
Lateral patella shift (%)	15.9 (9.9)	14.6 (9.2)	13.8 (6.8)	0.637 (−3.4–5.4)	0.647 (−4.6–2.9)
Patellar height measurement	PI group pre-surgery median (IQR)	PI group post-surgery median (IQR)	Control group median (IQR)	PI pre- vs post-surgery *p*-value^b^ (Z)	PI post-surgery vs control *p*-value^c^ (Z, U)
Insall–Salvati	1.4 (1.1–1.5)	1.2 (1.1–1.4)	1 (1–1.2)	0.019* (−2.35)	<0.001* (−4.20, 362)
Modified Insall–Salvati	1.9 (1.7–2.2)	1.8 (1.6–1.9)	1.6 (1.5–1.7)	0.003* (−2.97)	<0.001* (−3.54, 428)
Caton–Deschamps	1.1 (1.0–1.3)	1.0 (0.8–1.1)	0.9 (0.8–0.9)	<0.001* (−3.69)	0.006* (−2.75, 511)
Blackburne–Peel	1.0 (1.0–1.2)	0.9 (0.8–1.0)	0.8 (0.7–0.9)	<0.001* (−3.80)	0.001* (−3.23, 463)

*PI* patellofemoral instability, *SD* standard deviation, *CI* confidence interval, *IQR* interquartile range

*Statistically significant at <0.05 level

^a^Paired Student’s *t*-test

^b^Wilcoxon signed ranks test

^c^Mann–Whitney *U* test

## Discussion

The main findings of this study were that radiological parameters differed significantly between patients with PI and the Control group, and these parameters significantly improved in patients with PI following patellar stabilisation surgery. Disparities were found between measurements taken on MRI and X-ray across different skyline angles.

This is in line with current research in the field that shows radiological parameters differ significantly between patients with and without PI. A recent meta-analysis by White et al. [13] reported a significantly greater TT-TG, IS, and SA in patients with PI compared to the Control group. Whilst their study primarily focused on MRI, the present study observed the same difference between groups across both MRI and X-ray measurements. Furthermore, whereas White et al. [13] reported no CD disparity, the present study revealed significant differences between groups for all patellar height and patellofemoral measurements, with the exception of CA. These findings align with Yue et al. [29] who reported a significant difference between PI and Control groups for all 4 patellar height measurements, though they noted that patellar height measured greater on MRI compared to X-ray. Geraghty et al. [30] reinforced these findings, reporting significant differences between groups for BP, CD, and IS on both X-ray and MRI, with an increased TT-TG and SA observed on MRI. In contrast, MIS did not differ significantly between groups in their study. Despite the widespread acceptance of patellar height measurements for PI diagnosis, the optimal index of choice remains debated [11]. According to the literature, IS has been found to be the most commonly used method [13]. Verhulst et al. [31] found that IS yielded the best intra- and inter-observer reliability compared to other patellar height measurements on both X-ray and MRI. Similar observations were made by other studies in the field [13], with Yue et al. [29] reporting good reliability for CD on X-ray, and Geraghty et al. [30] reporting good reliability for CD and BP on both modalities. Although the present study showed all four measurements to be useful in the diagnosis of PI, it is not possible to comment on reliability as this was outside of the scope of the study.

The present study reported limited differences in patellofemoral measurements on X-ray between the PI group and Control group, with only SA and PTA being significantly different. This partially agrees with the findings of Dejour et al. [7] who reported a significant difference in SA between groups; however, they also found a difference in LPFA on X-ray imaging. Although PTA and LPFA are both used to quantify the degree of lateral patellofemoral tilt, the present study contrasts with that of Dejour et al. [7] in that no difference was found in LPFA. This may be explained by the lack of uniformity in the knee flexion angles at which X-rays were taken. Whilst optimal knee flexion for skyline X-rays is thought to be 30 degrees, Davies et al. [32] highlight that inconsistencies in knee flexion during imaging can lead to differences in measurements. Additionally, the relatively wide confidence intervals, particularly for CA and LPFA, may suggest limited precision of X-ray measurements in assessing patellofemoral alignment. In contrast, MRI measurements demonstrated significant differences between the PI and Control groups for all patellofemoral angles, with the exception of CA. These results are consistent with trends observed in the literature [3, 5].

Postoperative analysis demonstrated significant improvement for all patellar height measurements in the PI group following patellar stabilisation surgery. To our knowledge, this is the first study to comprehensively assess both patellar height and patellofemoral measurements, pre- and postoperatively, across a range of patellar stabilisation techniques in patients with PI. The findings of the present study align with other studies in the field [33–35]. Hashimoto et al. [33] found that both isolated MPFL and MPFL with TTT led to a significant improvement in IS postoperatively. Fabricant et al. [34] assessed patellar height measurements in adolescents following MPFL and observed a significant improvement in CD, IS, and MIS postoperatively. A similar observation was made in a recent meta-analysis by Knapik et al. [35], with CD and IS improving significantly following TTT in patients with patella alta. Together, these findings highlight the success of patellar stabilisation surgery in reducing patellar height and anatomical variations associated with PI.

This study demonstrated largely insignificant changes in patellofemoral measurements between pre- and postoperative groups, with the exception of SA significantly reducing from 143.4° (± 8.0°) to 139.5° (± 7.9°) (*p* = 0.005) following surgery. However, whilst the changes were not statistically significant for other measurements, the stabilisation surgery was still of clinical significance in reducing the PTA and LPS. This lack of significant change differs from the literature in which parameters are observed to change considerably. Hashimoto et al. [33] found CA to significantly improve following surgery. Similarly, Ercan et al. [36] showed the PTA to significantly improve from 23.4° (± 4.5º) to 12.5° (± 2.9°) (*p* < 0.05) and the CA to improve from 12.4° (± 8.9°) to −3° (± 4.6°) after MPFL surgery. However, for this study, the mean PTA and CA pre-surgery were within the parameters of normal, at 17.0° (± 6.3°) and −1.3° (± 26.1°) on a 30-degree skyline X-ray. Therefore, although a significant statistical change was not seen, it may have been due to such sizeable corrections not being required for the sample patient’s anatomy.

Furthermore, although patellar height measurements improved following surgery, the PI group continued to differ significantly from the Control group, indicating that whilst surgical intervention improves patellar height, it may not fully restore anatomy to that of stable knee joints. In terms of patellofemoral measurements, both the PTA and SA were significantly different preoperatively, but only the PTA (*p* = 0.048) remained significantly different postoperatively compared to the Control group. The normalisation of the SA suggests an improvement in the depth of the femoral trochlear groove, potentially contributing to more stable patellar tracking. However, the persistent significant differences in PTA may not reflect the clinically significant reduction observed from pre-surgery (17.0° ± 6.3°) to post**-**surgery (14.5° ± 4.0°), with the statistical significance between PI and Control groups being strongly so pre-surgery (*p* < 0.001), compared to only borderline statistical significance post-surgery (*p* = 0.048). Alternatively, it may be explained by limitations in radiographic sensitivity, as most preoperative measurements using MRI in the PI group were significantly different from those of the Control group.

This study reported significant differences in preoperative patellar height measurements (BP, CD, and MIS) between X-ray and MRI in the PI group. Comparable findings were reported by Yue et al. [29], who observed modality-based discrepancies across all four patellar height measurements, with MRI consistently yielding greater patellar height values. Similarly, patellofemoral measurements demonstrated significant preoperative differences between X-ray and MRI at various skyline views in the PI group. At 30 degrees, all measurements differed significantly (*p* < 0.05). At 60 degrees, significant differences were observed in LPS, PTA, and SA, whilst at 90 degrees, only LPS and PTA remained significantly different (*p* < 0.05). The appearance of an increased knee flexion angle in skyline measurements being more comparable to MRI may be due to the issue reported by Davies et al. [32], who noted that the fewest patellofemoral joint abnormalities were identified on 90-degree skyline X-rays. This may explain the differences observed between 30- and 90-degree comparisons to MRI. Overall, the differences in measurements between MRI and X-ray in this study reflect a poor association between modalities. MRI-based studies have demonstrated that previous pathological thresholds established for PI diagnosis on X-ray are not directly transferable to MRI [5]. Importantly, these findings have direct clinical implications. In clinical practice, patellofemoral imaging is frequently performed using MRI alone, without accompanying X-rays. Relying on X-ray derived thresholds in such cases may lead to misclassification or inaccurate estimation of patellar height and alignment. This further supports the need for established, modality-specific normative values and highlights an opportunity for future research to validate MRI-specific cutoffs that can support accurate diagnosis and clinical decision-making in PI. Recognising these differences, adjustments to diagnostic thresholds have been proposed when applied to MRI. Lee et al. [37] suggested increasing the cutoff values used for X-ray by 0.09–0.13 when assessing for patella alta or baja on MRI using the IS and BP indices. These adjustments are based on MRI’s ability to provide more accurate measurements due to the absence of superimposition of the lower trochlear structures that often complicate X-ray interpretation, as well as improved differentiation between osseous and cartilaginous structures [38]. Given these advantages, MRI has become a reliable and increasingly preferred modality for evaluating patellar height and patellofemoral alignment in the context of PI [5, 38].

A limitation of this study was the demographic difference between the Control group and the PI group, with PI typically affecting younger individuals, resulting in age discrepancies between groups. This was difficult to mitigate, as most other knee pathologies requiring imaging occur later in life. Additionally, the Control group presented with alternate pathology, suggesting their anatomy may not have been entirely normal. However, it was not ethically viable to include asymptomatic individuals, as imaging requires clinical justification. Measurement limitations arose due to patella alta and trochlear dysplasia. Patella alta limited the ability to visualise both the patella and trochlear groove on a single axial MRI slice, whilst trochlear dysplasia complicated angle measurements involving the trochlear groove’s contours. As outlined in the methodology, these were addressed through a literature review and the development of a new measurement approach. This technique allows key parameters such as patellar tilt to be quantified even when the patella and trochlear sulcus no longer appear on the same axial slice. By selecting the image where the patella appears at its greatest diameter, this method standardises the measurement plane. As a result, it may reduce slice selection bias, improve consistency between observers, and enable inclusion of severe patella alta cases that would otherwise be excluded. Although formal validation is still required, this approach offers the potential to improve the validity of patellofemoral MRI measurements and support inclusion of such cases in future research. Ideally, repeated measurements by multiple observers would allow assessment of inter- and intra-observer reliability. Despite these limitations, the study’s strengths include the comprehensive evaluation of multiple validated radiological measurements in patients with symptomatic patella instability and the introduction of newly described measurement techniques to help overcome difficult analysis in patients with grossly abnormal anatomy. It is also the first study to comprehensively assess both patellar height and patellofemoral measurements, pre- and postoperatively, across various surgical stabilisation techniques in patients with PI.

In conclusion, this study demonstrates that radiological parameters significantly differ between patients with PI and those with stable knees, with improvements observed following patellar stabilisation surgery. Patellar height emerged as the most consistent marker of instability, whilst discrepancies between MRI and X-ray measurements suggest the need for modality-specific normative values. These findings reinforce the value of radiological measurements as an important adjunct to physical examination in assessing PI and highlight the need for further standardisation to ensure consistency and enhance diagnostic accuracy.

## Data Availability

Data are available from the corresponding author on request.
